# Refractory hypoxia and saturation gap in a COVID-19 patient

**DOI:** 10.1016/j.plabm.2024.e00395

**Published:** 2024-04-18

**Authors:** Abidah Mobarak, Subashini C. Thambiah, Ana Daliela Masiman, Intan Nureslyna Samsudin, Yin Ye Lai

**Affiliations:** a Department of Pathology, Hospital Melaka, Ministry of Health Malaysia, Malaysia; b Department of Pathology, Faculty of Medicine & Health Sciences, Universiti Putra Malaysia, Malaysia

**Keywords:** Methemoglobinemia, COVID-19, Refractory hypoxia, Saturation gap

## Abstract

Acquired methemoglobinemia, predominantly due to oxidizing medications occurs when heme iron in hemoglobin is oxidized from ferrous to ferric ion and binds oxygen irreversibly leading to functional anemia, cyanosis, and tissue hypoxia. We report a case of a 60-year-old man with multiple comorbidities who was diagnosed with coronavirus disease 2019 (COVID-19) and developed methemoglobinemia after consumption of prescribed supplements. He presented with dyspnea and cyanosis. An oxygen saturation gap with characteristic chocolate-brown arterial blood indicated methemoglobinemia. Outsourced methemoglobin (MetHb) was increased at 9.0%. Despite aggressive intervention, he succumbed to his illness. In this case, we discuss the pathophysiology of why some individuals, especially the elderly with COVID-19 are more susceptible to develop methemoglobinemia after possibly being exposed to oxidizing agents. Laboratory methods for assessing oxygen saturation, including pulse oximetry, arterial blood gas and co-oximetry are examined in relation to this case. The importance of considering a diagnosis of methemoglobinemia based on clinical and biochemical findings although MetHb assay or co-oximetry are not readily available is also emphasized.

## Highlights

• Acute methemoglobinemia occurs when heme iron is oxidized from ferrous to ferric ion in Hb.
• Ferric iron is unable to bind and transport oxygen leading to functional anaemia, cyanosis, and tissue hypoxia.
• Causes of methemoglobinemia can be congenital or more commonly, acquired due to exposure to oxidizing substances.
• Refractory hypoxia, chocolate-brown arterial blood, and oxygen saturation gap are diagnostic clues.
• Disease severity in elderly COVID-19 patients is further exacerbated by the pro-inflammatory properties of MetHb.

## 1 Introduction

Acute methemoglobinemia occurs when heme iron is oxidized from ferrous (Fe^2+^) to ferric ion (Fe^3+^) in hemoglobin (Hb). Fe^3+^ is unable to bind and transport oxygen leading to functional anemia, cyanosis, and tissue hypoxia [1]. Causes of methemoglobinemia can be congenital [glucose-6-phosphate dehydrogenase (G6PD) deficiency, Hb M disease and congenital methemoglobinemia genotypes] or more commonly, acquired due to exposure to oxidizing substances such as phenazopyridine, benzocaine, dapsone and nitrates/nitrites [2].

Methemoglobinemia is a clinical diagnosis supported by refractory hypoxia, chocolate-brown arterial blood, and oxygen saturation gap. The gold standard for confirmation of methemoglobinemia is co-oximetry [3].

## 2 Case presentation

A 60-year-old man presented to the emergency department with a 5-day history of fever, shortness of breath, diarrhea, and lethargy. Three days prior to this, he was confirmed by reverse transcriptase-polymerase chain reaction for coronavirus disease 2019 (COVID-19) and started consuming ivermectin and the following supplements prescribed by his general practitioner: IMMNU3 GCG (glycine, cysteine, glutamine amino acids), REPosin capsule (curcumin, cinnamon) and CV Support Formula (combination of vitamins, minerals, enzymes, herbs including gingko biloba) [4]. He has a history of hypertension, dyslipidemia and diabetes mellitus on daily oral perindopril 4mg, ticlopidine 250mg, hydrochlorothiazide 25mg, slow k 600mg, fenofibrate 145mg, simvastatin 20mg and metformin 500mg twice daily.

On examination, he was afebrile, tachycardic, tachypneic with pallor and generalized cyanosis. Respiratory examination was unremarkable. Continuous pulse oximetry revealed 40% blood oxygen saturation (SpO_2_) at room air that increased to 50% on 15L oxygen using high-flow mask. Electrocardiography indicated sinus tachycardia, chest radiography showed borderline cardiomegaly and echocardiogram revealed dilated left atrial chamber with 45–50% ejection fraction. He further deteriorated requiring intubation and mechanical ventilation.

His arterial blood sample color was chocolate-brown (Fig. 1a). Full blood count revealed anemia, raised white cell count with lymphocytosis and neutrophilia. Anemia, urobilinogenuria 2+ and marked anisocytosis with presence of bite and blister cells on peripheral blood film suggested oxidant-induced hemolytic anemia. Increased urea 34.4mmol/L, creatinine 533μmol/L, potassium 5.6mmol/L and hematuria [5+ red blood cells (RBC) on urinalysis] indicated acute kidney injury (AKI). Hepatocellular injury was evident with elevated total bilirubin 33μmol/L, liver transaminases (aspartate aminotransferase 597U/L; alanine aminotransferase 147U/L) and lactate dehydrogenase (LDH) 4447U/L. Normal G6PD level ruled out G6PD deficiency as a cause for the non-immune hemolytic anemia (Table 1).

**Fig. 1a:**
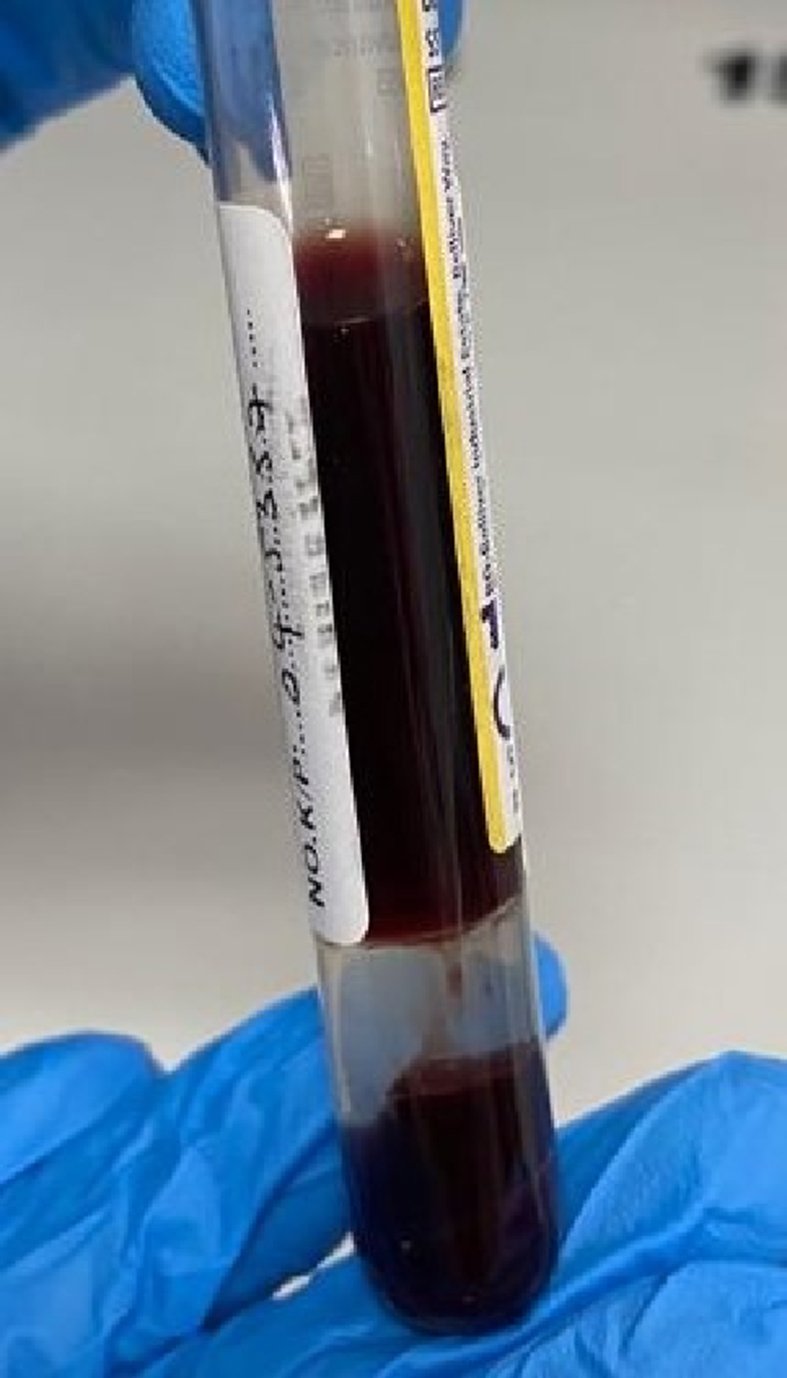
Chocolate-brown colored arterial blood of the patient. (For interpretation of the references to color in this figure legend, the reader is referred to the Web version of this article.)

**Table 1:** Laboratory investigation results.

Parameters	Reference interval	On admission	Days after admission		
			Day 1	Day 2	Day 3
**Hematology**					
Hb, g/L	130–170	71	69	88	93
Hct, %	40–50	15.9	15.6	23.3	26.4
WBC, 10^9^/L	4.0–10.0	29.5	31.4	63.4	81.6
Neutrophils,10^9^/L	2.0–7.0	24.2	25.6	29.5	76.1
Lymphocytes, 10^9^/L	1.0–3.0	3.5	5.4	2.6	0.1
Plt, 10^9^/L	150–410	260	266	300	383
Rtc, %	0.5–2.5	17.7 %			
G6PD (Fluorescent spot test)		Normal			
**Clinical Chemistry**					
Urea, mmol/L	2.5–7.8	34.4	40.9	47.2	48.1
Na, mmol/L	133–146	130	140	135	134
K, mmol/L	3.5–5.3	5.6	4.4	5.5	7.5
CREA, μmol/L	59–104	533 (*baseline 119* 7 months *ago)*	606	691	794
TP, g/L	66–83	69	68	67	61
Alb, g/L	32–48	41	39	34	35
AST, U/L	0–34	597	604	892	701
ALT, U/L	10–49	147	167	153	154
ALP, U/L	46–116	47	52	77	75
TBIL, μmol/L	0–21	33.2	25.1	36.8	50.1
DBIL, μmol/L	≤5.1	17.7		21.2	31.5
LDH, U/L	120–246	4447	>4500	>4500	>4500
CRP, mg/L	<10			231.4	287.8
CK, U/L	46–171		286	423	
Ferritin, pmol/L	48–708	>36300	>36300	>36300	
Glc (random), mmol/L	<11.1			14.3	
MetHb level, %				9.0 *(post-blood transfusion)*	
ABG					
pH	7.35–7.45	7.17	7.31	7.32	7.23
pO_2_, kPa	11.07–14.40	75.06	50.53	15.73	12.00
pCO_2_, kPa	4.67–6.40	3.90	4.00	6.13	4.30
HCO_3_^−^, mmol/L	22–26	10.6	18.6	25.2	13.4
Urinalysis			RBC 5+		
			Bilirubin -ve		
			Urobilinogen 2+		
			Ketone 1+		
			Leucocyte -ve		
			Nitrite -ve		

Hb – hemoglobin, Hct – hematocrit, WBC - white blood cells, Plt – platelets.

Rtc – reticulocytes, G6PD – glucose-6-phosphate dehydrogenase, Na – sodium.

K – potassium, CREA – creatinine, TP – total protein, Alb – albumin.

AST – aspartate aminotransferase, ALT – alanine aminotransferase, ALP – alkaline phosphatase, TBIL – total bilirubin, DBIL – direct (conjugated) bilirubin, LD – lactate dehydrogenase, CRP - c-reactive protein, CK -creatine kinase, Glc – glucose.

MetHb – methemoglobin, ABG – arterial blood gas, pO_2_ – partial pressure oxygen, pCO_2_ – partial pressure carbon dioxide, HCO_3_^−^ - bicarbonate, RBC – red blood cells, -ve – negative.

As the patient's consciousness level was deteriorating, non-invasive positive-pressure ventilation (NPPV) with bilevel airway pressure was initiated. ABG demonstrated metabolic acidosis (Table 1) with calculated oxygen saturation (SaO_2_) at 100%. Nonetheless, there was discrepancy between his continuous pulse oximeter readings that showed SpO_2_ of 50% and ABG analysis under NPPV showing paradoxical SaO_2_ of 100% and partial pressure of oxygen (PaO_2_) of 563mmHg (reference interval, 83–108).

Metabolic acidosis was attributed to AKI. Hyponatremia (sodium 130mmol/L) was attributed to AKI and possibly syndrome of inappropriate antidiuresis due to the central effect of interleukin-6 that stimulates the release of vasopressin in COVID-19 [5]. Serum ferritin and LDH levels were markedly elevated. Ferritin is an acute phase reactant. Release of pro-inflammatory cytokines, cellular damage, metabolic acidosis, associated reactive oxygen species generation and secondary tissue damage are hypothesized mechanisms of high serum ferritin level in COVID-19 [6]. LDH is an intracellular enzyme that catalyzes the conversion of pyruvate to lactate in the absence of oxygen in last step of glycolysis. Elevated LDH levels indicate tissue hypoperfusion, suggesting cellular damage, hypoxia, or cell death [7,8]. Systematic reviews and meta-analysis studies have demonstrated that both serum ferritin [9,10] and LDH [7,8] are associated with disease severity and poor prognosis in patients with COVID 19. The patient was admitted to intensive care unit for further management of Stage 5 COVID-19 [11].

He was transfused two pints of packed RBC. The persistent chocolate-brown arterial blood, low SpO_2_ despite mechanical ventilation with 100% fraction of inspired oxygen and paradoxical SaO_2_/PaO_2_ levels suggested methemoglobinemia. As methemoglobin (MetHb) testing was not available on site, the test was outsourced on day 2 of admission and reported as 9.0%. He was then started on intravenous methylene blue 100mg (about 1mg/kg) over 15 minutes, insulin, tazocin, dexamethasone and oral azithromycin. Subsequent laboratory workup showed no improvement (Table 1). The SpO_2_ did not improve, and his renal function further declined requiring dialysis. Another cycle of packed RBC was transfused. Unfortunately, he succumbed to his illness due to multi-organ failure.

## 3 Discussion

We present a 60-year-old man with multiple comorbidities and COVID-19 who presented with acute hypoxia after consumption of supplements and was found to have chocolate-brown arterial blood with discordant SpO_2_ and SaO_2_/PaO_2_ values leading to a diagnosis of methemoglobinemia. Acute methemoglobinemia causes functional anemia, cyanosis and tissue hypoxia when heme iron is oxidized from Fe^2+^ to Fe^3+^ in Hb, preventing the heme moiety from carrying oxygen. This leads to a left shift in the oxygen dissociation curve causing decreased tissue oxygen release [1].

As this patient presented acutely after the consumption of various supplements, he was treated as drug-induced methemoglobinemia though the causative agent was not identified. Interestingly, gingko biloba leaf extract exhibits a dual effect on RBC, acting both protectively and disruptively, depending on the presence of external stressors. Its disruptive action becomes apparent at higher doses, where it damages RBC by increasing their fragility, altering cellular structure, and triggering glutathione consumption which may lead to MetHb formation [12]. Nevertheless, further investigation and research are necessary to thoroughly explore the proposed mechanism. In addition, methemoglobinemia can also be induced by high-nitrate foods such as beets, spinach, carrots, borage (an edible flowering plant), and chard (a leafy green vegetable) [13]. However, in this case, the specific dosage of supplements consumed, and his dietary intake or drug history are not fully known. Additionally, not every individual exposed to oxidizing agents develops methemoglobinemia, highlighting the variability in metabolism among patients [1] and possibly diverse contributing factors. Unfortunately, due to limited resources, no further analysis of the supplements or urine toxicology analysis were available.

In this patient, however, other factors may have increased his susceptibility to develop methemoglobinemia for example coronavirus related Hb alterations. Several proteins of the coronavirus can attach to the porphyrin of Hb, altering its oxygen-binding capacity and hence, lowering oxygen release in tissues [14]. This patient's age with multiple comorbidities prior to the onset of illness would have further contributed to the severity of methemoglobinemia in COVID-19 infection. In elderly COVID-19 patients, the degree of critical illness combined with comorbidities causes greater oxidative stress and thus, increases the susceptibility of RBC to drug-induced methemoglobinemia. Aged cells are more prone to oxidation as they cannot detoxify drugs or chemicals as efficiently as younger cells [15]. Moreover, in a septic patient, the release of nitric oxide which converts to nitrate and ultimately to MetHb may also cause methemoglobinemia [16]. Anemia as part of the physiological response to infection or ongoing systemic inflammatory reaction is known as “anemia of inflammation.” Acute anemia may also lead to formation of MetHb as a by-product of a physiological reaction due to an adaptive increased nitric oxide signaling. Disease severity is further exacerbated by the pro-inflammatory properties of MetHb [15].

Pulse oximetry relies on the principle that oxyhemoglobin and deoxyhemoglobin exhibit differential absorption of red light at 660nm and near-infrared (IR) light at 940nm [17]. The SpO_2_ is determined from the ratio of the absorbance at these two wavelengths. MetHb disrupts this ratio by equally absorbing light at both wavelengths. The pulse oximeter converts the calculated oxyhemoglobin to deoxyhemoglobin ratio into SpO_2_, where a ratio of 1 corresponds to a SpO_2_ of approximately 85%. Therefore, as the concentration of MetHb in the blood surpasses 35%, the SpO_2_ is expected to stabilize around 85% [18]. In this patient, however, continuous pulse oximetry revealed a SpO_2_ of 40% (room air) to 50% (on 15L oxygen). This is significantly lower than the anticipated SpO_2_ plateau of 85% in methemoglobinemia, suggesting the presence of co-existing underlying pathology in this patient.

A SpO_2_ reading of 40% generally indicates severe hypoxemia. Potential causes in this patient include moderate anemia (Hb 71g/L) and sepsis with hypoperfusion. It is crucial to note that anemia itself does not cause a spuriously low SpO_2_ but may lead to an underestimation of SpO_2_ in individuals with true hypoxemia, without significantly affecting SpO_2_ measurements in normoxic individuals. This is because the simplified Beer-Lambert equation, which assumes a single, well-defined light path, does not account for red and near-IR light scatter by human tissues and calibration curves are derived from healthy individuals. In anemic conditions, with fewer RBC, there is less light scatter, altering the pathlength for transmitted red and near-IR light, resulting in lower SpO_2_ readings [17].

Studies present conflicting findings regarding how SpO_2_ readings are affected in the context of sepsis and septic shock. One proposed hypothesis for this inconsistency is that vasodilation induced by sepsis leads to the formation of arterio-venous shunts. These shunts result in venous pulsations, causing the pulse oximeter to spuriously detect some venous blood as arterial. The significant variation in venous volume with each cardiac cycle due to venous pulsations can contribute to a falsely low SpO_2_ reading [17].

MetHb has a half-life of 55 minutes with levels below 2% in normal physiologic states [18]. The severity of clinical features in methemoglobinemia is dependent upon the proportion of MetHb, the rate at which MetHb levels rise, the inherent capacity of the patient to eliminate it, and the patient's underlying functional condition. MetHb can be quantified either in terms of concentration or percentage, with the percentage derived by dividing the concentration of MetHb by the total Hb concentration [19].

Cyanosis induced by MetHb becomes clinically noticeable when the MetHb level reaches 15g/L, typically representing around 10–15% of total Hb in a normal individual. MetHb levels exceeding 70% can lead to fatality. However, interpreting MetHb levels in relation to symptoms can be challenging, as it is generally expressed as a percentage of total Hb [20]. For instance, a MetHb concentration of 15g/L may constitute 10% in a healthy individual with a baseline Hb of 150g/L. In contrast, the same MetHb concentration in an anemic patient, as in this case with a baseline Hb of 71g/L, would represent a higher percentage of 21.13%. The former may remain asymptomatic with a functional Hb concentration of 135g/L, while the latter, with a functional Hb concentration of 56g/L, could experience severe symptoms at a MetHb level of 21% due to reduced ability of functional Hb to release oxygen [20]. The coexisting anemia and sepsis in this patient most likely exacerbated methemoglobinemia symptoms by impeding oxygen delivery to tissues [20]. Therefore, assuming the patient's MetHb was 15g/L on admission, the patient's pre-transfusion MetHb level was approximately 21%, corresponding to his hypoxic state clinically. The subsequent decrease in MetHb to 9.0% was attributed to blood transfusion, as the introduction of donor RBC into circulation increases the concentration of functional Hb, thereby reducing the calculated MetHb concentration in blood [14].

The definitive diagnosis of methemoglobinemia can be confirmed using the gold standard, co-oximetry. Co-oximetry, akin to pulse oximetry, measures light absorbance at 660nm and 940nm. However, it offers an additional advantage by measuring light absorbance at wavelengths of 600nm and 631nm. Consequently, co-oximetry provides clinicians with a non-invasive method to directly assess MetHb levels, as MetHb specifically absorbs light at the 631nm wavelength [21].

In settings where MetHb assay or co-oximetry is not readily accessible, maintaining a high index of clinical suspicion for methemoglobinemia is crucial, and treatment should not be delayed. In this case, the patient received intravenous methylene blue, the primary antidote for methemoglobinemia, only after obtaining the MetHb result on day 2 of admission. Diagnostic indicators for methemoglobinemia in this patient included the observation of chocolate-brown arterial blood (Fig. 1a) that did not transition to a bright red color (Fig. 1b) upon exposure to air, refractory hypoxia (cyanosis unresponsive to 100% oxygen), and a disparity between the low measured SpO_2_ on pulse oximetry and the elevated SaO_2_ calculated on ABG, resulting in an abnormal oxygen saturation gap exceeding 5%. The calculated SaO_2_ from ABG is derived from PaO_2_ and pH. In methemoglobinemia, where PaO_2_ remains within normal limits, this leads to a calculated SaO_2_ that is normal but inaccurate [18].

**Fig. 1b:**
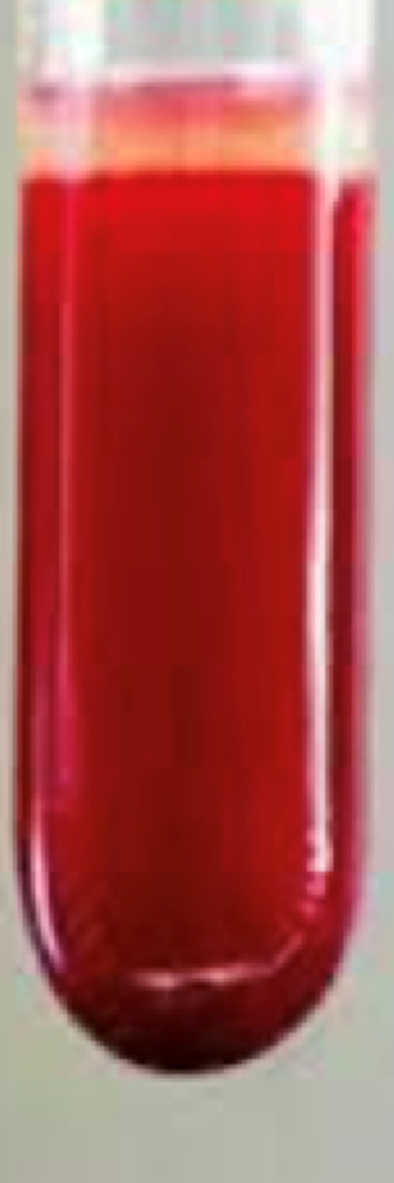
Bright red colored arterial blood of a healthy individual. (For interpretation of the references to color in this figure legend, the reader is referred to the Web version of this article.)

Methylene blue is contraindicated in patients with known G6PD deficiency, which was ruled out in this patient. This is because methylene blue has an oxidant potential and depends upon nicotinamide adenine dinucleotide phosphate generated by G6PD in the reduction process of MetHb; hence may induce hemolysis in G6PD deficient patients [1]. In cases of refractory hypoxia, red cell exchange transfusion may be beneficial due to removal of RBC destruction products and Hb from the circulation [14]. This patient, however, passed away before any further intervention could be carried out.

## 4 Conclusion

In a critically ill COVID-19 patient, presenting with refractory hypoxia, chocolate-brown arterial blood, and oxygen saturation gap exceeding 5%, a diagnosis of methemoglobinemia should never be missed. This case report emphasizes the importance of considering methemoglobinemia based on clinical and biochemical findings although MetHb assay or co-oximetry are not readily available. Early detection allows for timely intervention and better prognosis.

## Patient consent

Informed consent was sought from the next-of-kin of the deceased patient for publication of the case report and accompanying image.

## CRediT authorship contribution statement

**Abidah Mobarak:** Writing – review & editing, Writing – original draft, Investigation, Formal analysis, Conceptualization. **Subashini C. Thambiah:** Writing – review & editing, Writing – original draft, Supervision, Investigation, Formal analysis, Conceptualization. **Ana Daliela Masiman:** Writing – review & editing, Supervision, Conceptualization. **Intan Nureslyna Samsudin:** Writing – review & editing, Investigation. **Yin Ye Lai:** Writing – review & editing, Investigation.

## Declaration of competing interest

The authors declare that they have no known competing financial interests or personal relationships that could have appeared to influence the work reported in this paper.

## Data Availability

No data was used for the research described in the article.
